# Inpatient satisfaction with medical information received from caregivers: an observational study on the effect of social deprivation

**DOI:** 10.1186/s12913-017-2728-8

**Published:** 2017-11-23

**Authors:** L. Moret, E. Anthoine, A. Pourreau, F. Beaudeau, B. Leclère

**Affiliations:** 1 Public Health Department, University Hospital, Saint-Jacques Hospital, 85, rue Saint-Jacques, 44093 Nantes Cedex, France; 2 UMR 1246 INSERM SPHERE “MethodS in Patients-centered outcomes and HEalth ResEarch”, University of Nantes, Bd Benoni-Goullin, 44200 Nantes, France; 3 BIOEPAR, INRA, Oniris, 44307 Nantes, France

**Keywords:** Inpatient satisfaction, Medical information, Shared decision-making, Social deprivation

## Abstract

**Background:**

The main objective of this study was to explore the relationships between inpatients’ social differentiation and satisfaction with the medical information delivered by caregivers.

**Methods:**

In four departments of a teaching hospital, patients were enrolled as well as their attending physician and one of the nurses assigned to them. Structured survey questionnaires were administered face-to-face to patients and caregivers. Patients were asked to rate their satisfaction with the medical information received, the quality and duration of the interactions with the caregivers, and their experience regarding their involvement in medical decision-making. Caregivers were asked to rate their perception of the patients’ social position and involvement in medical decision-making. Social deprivation was assessed using the EPICES score in particular. The statistical analysis was mainly descriptive and completed by a structural equation model.

**Results:**

A sample of 255 patients, 221 pairs of patient-physician and 235 pairs of patient-nurse were considered. One third of the patients (32.7%) were identified as socially deprived. They were significantly less satisfied with the information they received on their health status or their treatment; 56.7% of patients thought that they received sufficient explanations without having to ask. This proportion was significantly lower in socially deprived patients (42.3%) compared to not deprived patients (63.6%, *p* < 0.01). Patients’ reported involvement in medical decision-making was significantly lower for socially deprived patients (75.0% vs 89.0%, *p* < 0.001). The structural equation model showed that the main determinant of patients’ satisfaction regarding medical information was their perceived involvement in informed medical decision-making (CFI = 0.998, RMSEA = 0.022).

**Conclusions:**

These findings suggest that physicians and nurses need training on communication targeted towards vulnerable patients, in order to improve the accessibility of medical information, and thus to reduce health inequalities.

## Background

Patient information is one of the key elements of the physician-patient relationship [1–4]. It has been shown to be strongly linked to patients’ satisfaction and compliance, but also to morbidity measures [5, 6]. Among the existing models of interaction, the traditional asymmetric physician-patient relationship—the dominant “paternalistic model”—is slowly being replaced by more patient-centred models, based on negotiation and cooperation. For example, Street et al. highlighted the importance of a shared identity between patient and physician, combining reciprocity and mutual influence [3, 7]. It has also been shown that patients taking an active role during physician-patient interactions were more inclined to ask questions, understand medical explanations and make good decisions about their care [8, 9].

However, the physician-patient relationship still usually remains unequal, which can induce health inequalities. First, physicians’ beliefs about their patients can lead to disparities in treatment [10]. Second, the interactions between physicians and patients seem to vary according to their perceived social distance [11–14]. Several studies showed that patients from lower social classes receive significantly less information and are significantly less involved in shared decision-making [15–18]. Indeed, these patients are usually less proactive in eliciting information and their physicians frequently underestimate their need for it. For example, it has been shown that physicians are less likely to discuss cancer screening tests or post-mastectomy reconstruction with patients who had a lower education level [16, 18]. In the United States, where ethnicity and socioeconomic status are strongly correlated, African-American patients seem to receive less information from their physician after an angiography [15] or in the context of kidney transplantation [19].

Most of the published studies have explored these interactions during medical encounters, but we hypothesize that this inequality in patients’ information also occurs in other contexts, especially within hospitals, where patients usually interact with several caregivers and not solely their attending physician. In this context, disagreements between health care professionals concerning their respective roles in information delivery may also occur. In a study conducted by our team, nurses considered that providing patients with information was an integral part of their mission (giving explanations during care provision, relaying, re-explaining and completing the information given by the physicians). However, nurses also reported that they usually did not know what the patients were told by the physicians, which complicated their task and hindered the quality of the information delivered. Physicians, in contrast, had a much more restrictive view of patient information, only considering it during a face-to-face interview [20].

The main objective of this study was to explore the relationship between inpatients’ social differentiation and their satisfaction with the medical information delivered by the caregivers. The secondary objectives were to explore the relationship of inpatients’ social differentiation with their satisfaction regarding their interactions with the caregivers, and with their experience regarding their involvement in shared decision-making.

## Methods

### Ethical and consent considerations

The research protocol was approved by the “Groupe nantais d'éthique dans le domaine de la santé” (Nantes ethics committee in health research). All eligible inpatients, physicians and nurses were invited to participate by the interviewer and gave their verbal informed consent before the interview. The data were anonymized before recording. According to the L1121–1 and R1121–2 articles of the French code of public health, written consent and IRB approval are not necessary for non-interventional research.

### Study population and setting

This study was conducted during the second semester of 2011 at the Nantes University Hospital (France). It involved 10 voluntary units from two surgery departments (gynaecology and orthopaedic surgery) and two medicine departments (internal medicine and emergency medicine). Inpatients were recruited 24 h before discharge. To ensure the inclusion of enough socially deprived patients, that means patients who cannot have an easy and frequent access to the many different aspects of their culture and society, due to a combination of factors such as low socioeconomic status or poor education, the minimum sample size to be considered was 172, under the assumption that socially deprived patients represented 35% of the whole population of inpatients [21]. A total of 333 patients fulfilled the eligibility criteria for inclusion (age ≥ 18, proficiency in French, hospitalization ≥3 days, informed consent given), but 47 of them left the hospital before being interviewed, and 31 finally refused to participate. Therefore, 255 patients actually participated (participation rate: 76.6%). To ensure a high response rate, patient questionnaires were completed by trained interviewers during a face-to-face interview of 15 to 30 min.

For each patient, the attending physician and one of the assigned nurses were also recruited. Once the patients were interviewed, their attending physician and nurse were contacted and interviewed face-to-face, on the day of discharge or within the next 2 days. The interviews lasted from 2 min for nurses to 5 min for physicians. Every caregiver accepted to participate, but some of them were not available at the time of interview and some patients did not have a designated attending physician. The final sample consisted of 221 patient-physician and 235 patient-nurse pairs (i.e. respectively 86.7 and 92.2% of the patient sample).

### Measures

#### Patient questionnaire

##### Patients’ status towards social deprivation

The patients’ deprivation status was assessed in two ways by the patient himself or herself: an objective measure using a standardised score and a self-perceived measure. The individual index used to objectively measure the level of deprivation was the EPICES score (“Evaluation de la Précarité et des Inégalités de santé dans les Centres d’Examens de Santé” - Evaluation of Deprivation and Inequalities in Health Examination Centers). This score was validated in 2002 on a cohort of 197,389 subjects examined in 58 French health examination centers [21, 22]. It consists of 11 items related to isolation (one item), health insurance status (one item), economic status (three items), social support (three items) and leisure activity (three items) (Table 1). For each item, a binary response Yes/No is expected from the patient. The total EPICES scores range from 0 to 100, from the lowest to the highest social deprivation. Even though this score can be considered as continuous, it has been mostly used with a threshold to categorize patients’ deprivation status [22–24]: patients were considered deprived if their score is superior to 30.17 (which correspond to the fourth quintile of the score distribution in the original study). In Table 2, deprived patients were identified using this threshold.

**Table 1:** Description of the 11 EPICES items scale. For each item, a binary response Yes/No is expected from the patient

1. Do you sometimes meet with a social worker (welfare worker, educator)?
2. Do you have complementary health insurance (mutual insurance)?
3. Do you live as a couple?
4. Are you a homeowner or will you be one in the near future?
5. Are there periods in the month when you have real financial difficulties in facing you needs (food, rent, electricity)?
6. Have you participated in any sports activities in the last 12 months?
7. Have you gone to any shows (cinema, theatre) in the last 12 months?
8. Have you gone on holiday during the past 12 months?
9. Have you seen any family members in the past 6 months (other than your parents or children)?
10. Did you have difficulties (financial, family or health), is there anyone around you who could take you in for a few days?
11. Did you have difficulties (financial, family or health), is there anyone around you who could help you financially (material aid such as lending you money)?

**Table 2:** Main patient’s characteristics (*n* = 255)

Patients’ characteristics	Patients			*p*
	Whole sample (%)	Deprived^a^ (%)	Not deprived (%)	
Gender				
Male	34.5	34.6	33.5	NS**
Female	65.5	65.4	67.5	
Age group (years)				
≥ 65	43.1	59.3	34.7	<0.01
< 65	56.9	40.7	65.3	
Educational level				
High	25.9	14.8	32.3	<0.01
Medium	38.4	35.8	39.5	
Low	35.7	49.4	28.2	
Employment status				
Employed active	30.6	16.0	37.7	<0.01
Retired	50.4	60.5	45.5	
Other inactive	19.0	23.5	16.8	
Having a referring GP	97.6	95.1	99.4	<0.05
Chronically diseased or handicapped				
Yes	65.9	71.6	63.4	NS
No	34.1	28.4	36.6	
Perceived health status (compared to people of the same age)				
Lower	34.0	31.2	36.9	NS
Similar or better	66.0	68.8	63.1	

**NS non-significant (*p* > 0.05)

^a^EPICES score > 30.17 on a scale from 0 to 100

In addition to this objective measure, the patients were also asked to rate their perceived social status on a scale from 1 to 10.

##### Patients’ satisfaction about the quality of the medical information they received from caregivers

Patients’ levels of satisfaction with the medical information they received (about health status, treatment, investigations and discharge) and with the quality and duration of their interactions with the caregivers were measured using 5 answer modalities (very satisfied, satisfied, moderately satisfied, dissatisfied, extremely dissatisfied).

##### Patients’ perceived experience concerning their involvement in informed medical decision-making

The patient were also asked if they felt that they were involved in medical decision-making and if they received sufficient information without having to ask. Five answer modalities were available: always, almost always, often, sometimes and never.

Finally, the questionnaire included descriptive demographic and socioeconomic variables: age, gender, educational level (high: high-school graduation and higher; medium: middle-school graduation; low: no formal diploma), employment status (employed active, retired, other inactive), declaration of a referring general practitioner (yes/no), existence of a chronic disease or handicap (yes/no).

#### Questionnaire administrated to caregivers

Physicians and nurses were asked separately to answer a specific questionnaire about their perception of their patient’s social position on a scale from 1 to 10, and about their perception on how the patient was involved in the medical decision making through the same 5 modalities as patients: always, almost always, often, sometimes and never.

### Statistical analysis

Traditional descriptive statistics (frequency, median, mean ± standard deviation) were used to describe the sample. The characteristics of deprived and not deprived patients were then compared by Chi-squared tests for categorical variables, trend Chi-squared test for ordinal variables and by T-tests or paired T-tests for continuous variables. Patients and caregivers’ perception were compared considering the patient-nurse and patient-physician pairs separately.

As many of the measured variables were supposedly correlated, we chose to model the relationships between them with a structural equation model. First, we studied all pairwise Spearman correlation coefficients (ρ) of the variables deemed as relevant based on the results of the bivariate analyses. The most correlated variables were grouped into latent factors that were used as independent variables in the final model. Diagonally weighted least squares were used to estimate the model parameters, and the full weight matrix was used to compute robust standard errors. Model selection was based on the comparative fit index (CFI) and the root mean square error of approximation (RMSEA).

All the analyses were performed using R 3.0.2. and IBM SPSS Statistics 19. Structural equation modelling was performed in R using the lavaan package version 0.5–20 [25].

## Results

### Characteristics of the sample

The characteristics of the patient sample are described in Table 2. Two-thirds of the patients were females; the mean age was 59.1 years (SD = 21.4; median of 60). Half of the patients were retired and nearly two thirds were chronically ill or handicapped. Most of them (93%) designated French as their mother tongue.

**Fig. 1:**
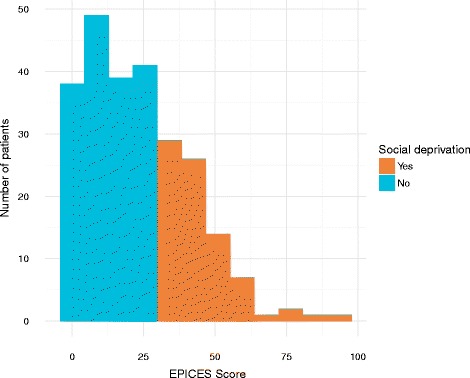
Histogram of the EPICES score in the study sample (*n* = 248)

The distribution of the EPICES score in the sample is displayed in Fig. 1. This score was available for 248 patients. Overall, almost a third of them (*n* = 81; 32.7%) were identified as socially deprived. This proportion varied from 22% in the surgery departments to 52% in the emergency medicine department. Table 1 shows the description of the 11 EPICES items scale.

Comparisons between deprived and not deprived patients are available in Table 2. Deprived patients were significantly older, had a lower educational level and were more often retired than patients who were not socially deprived. In contrast, no significant differences were observed regarding chronic disease and handicap, and perceived health between the two groups.

### Comparisons between caregivers’ and patients’ perceptions of patient social level

The average patients’ self-perceived social level was 6.1 (SD = 1.5) out of 10 for the whole sample. Deprived patients gave on average significantly lower ratings than not deprived patients (5.7 (SD = 1.7) vs. 6.3 (SD = 1.4); *p* < 0.01). For the whole sample, the patients’ perceived social level, as rated by the physicians and the nurses, averaged 6.5 (SD = 2.0) and 6.5 (SD = 1.6) out of 10 respectively. On average, the caregivers’ ratings were significantly higher than the patients’ self-ratings (*p* < 0.001), with differences of +1.11 for the physicians and +1.37 for the nurses. Physicians and nurses gave significantly (*p* < 0.001) lower ratings to deprived patients (5.3 (SD = 2.3) and 5.9 (SD = 1.8) respectively) than to not deprived patients (7.0 (SD = 1.6) and 6.8 (SD = 1.4) respectively).

### Patients’ satisfaction about the quality of the medical information they received from caregivers

**Fig. 2:**
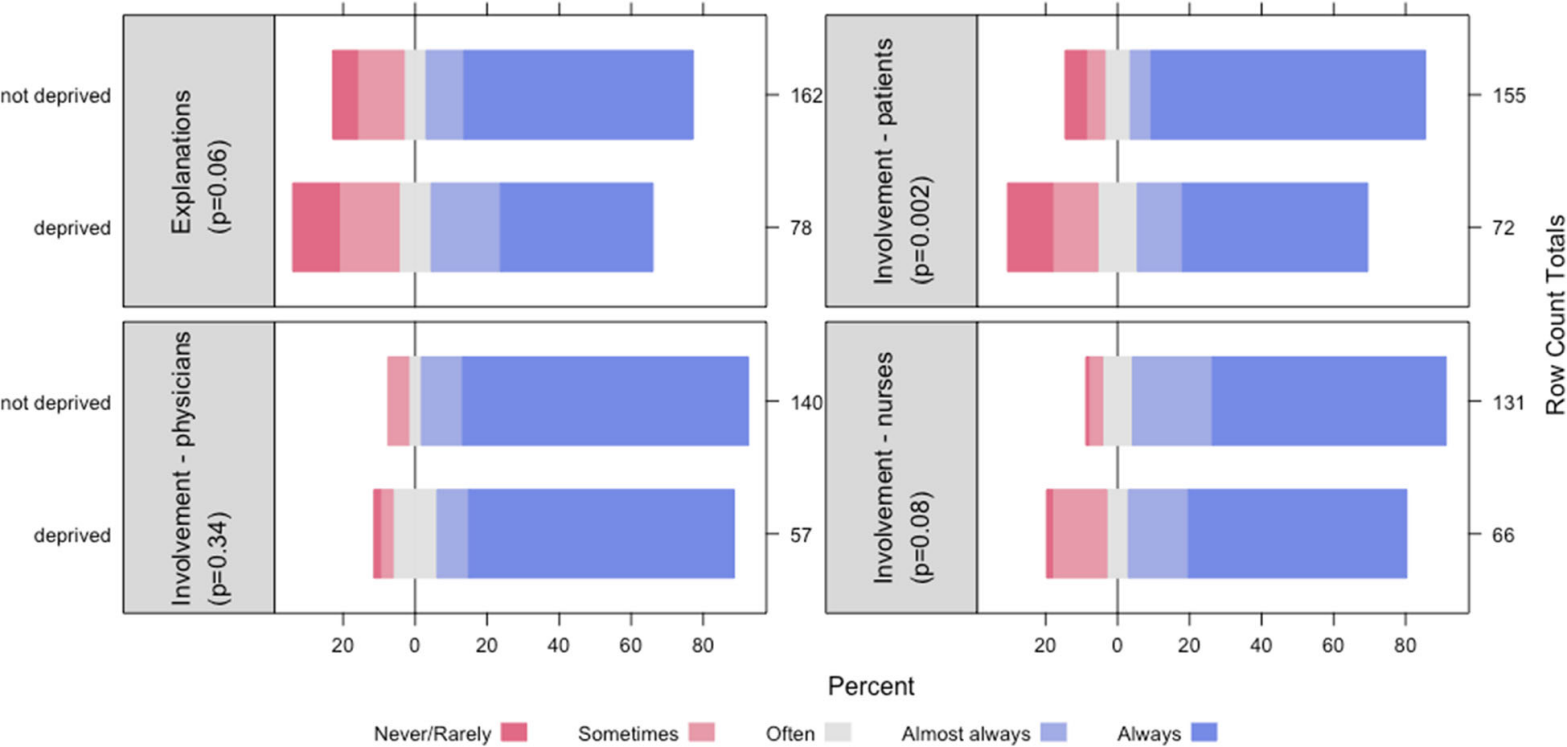
Comparison of deprived and not deprived patients’ satisfaction with medical information received. *: Significant difference between deprived and not deprived patients (Trend chi squared)

More than 85% of the patients were satisfied with the medical information given by the caregivers (Fig. 2). Socially deprived patients were significantly less satisfied with the information they received on their health status or their treatment (*p* < 0.05). The quality of their care was perceived as excellent or very good by 61.4% of the patients, regardless they were deprived or not deprived (trend *p*-value: 0.57).

### Patients’ perceived involvement in medical decision making

**Fig. 3:**
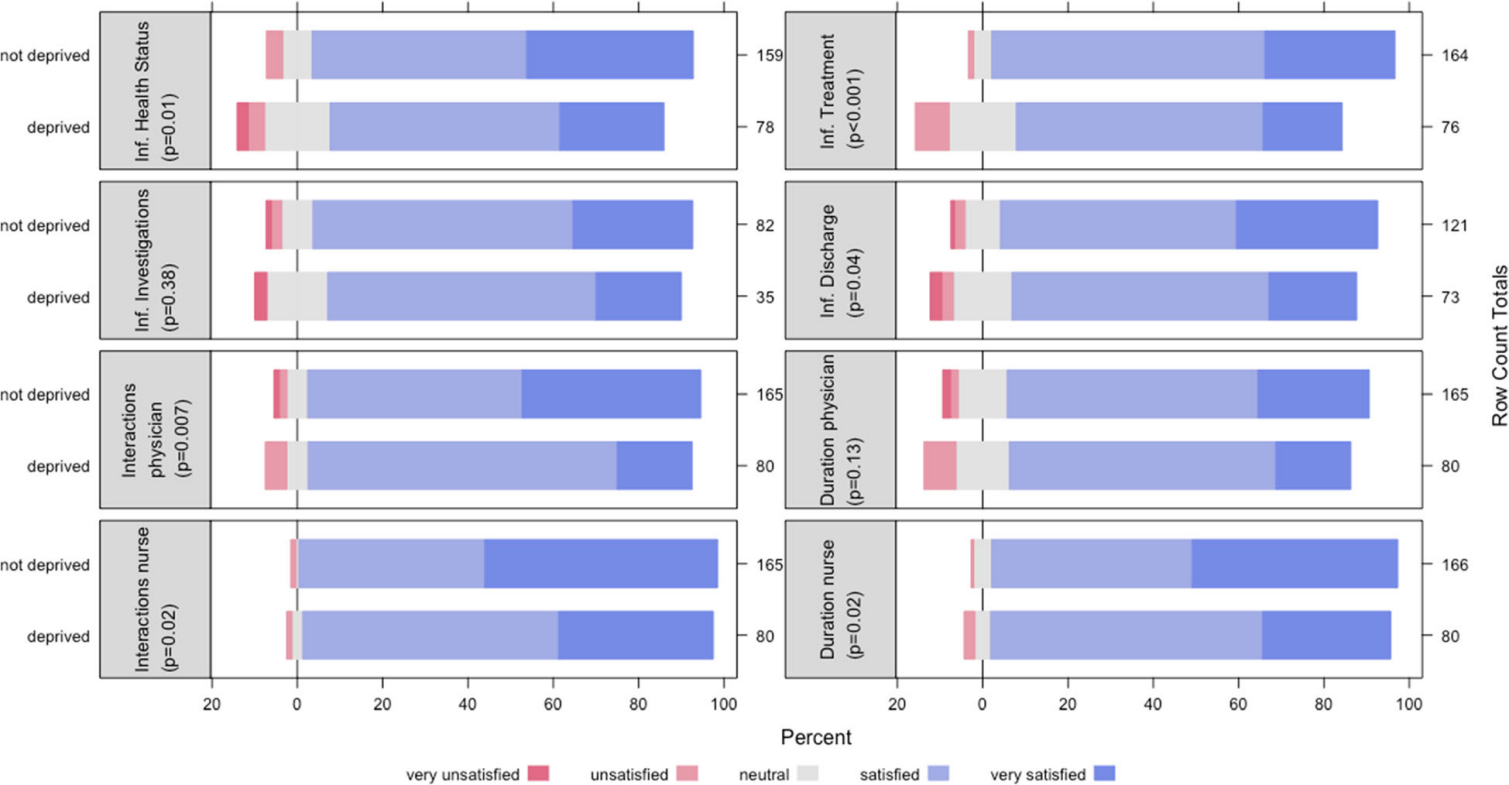
Comparison of deprived and not deprived patients’ experience concerning their involvement in the medical decision. *: Significant difference between deprived and not deprived patients (Trend chi squared)

Overall, only 56.7% of patients thought that they received enough explanations without having to ask for them. These results were significantly lower for socially deprived patients (42.3% vs. 63.6%, *p* < 0.01) (Fig. 3). More than 80% of patients perceived that they had always, almost always or often been involved in medical decision-making. This proportion was significantly lower for socially deprived patients (75.0% vs. 89.0%, *p* < 0.001) (Fig. 3).

In comparison, the proportion of patients having always or almost always been involved in medical-decision making was estimated at 83.7% by nurses and at 88.3% by physicians, with no significant difference between deprived and not deprived patients (trend *p*-values: 0.08 for nurses, 0.34 for physicians).

### Structural equation modelling

The study of the Spearman’s coefficients showed 1) strong correlations between the duration and the perceived quality of the interactions with caregivers (ρ = 0.86 and 0.75 for nurses and physicians respectively); 2) a moderate correlation between the two variables relating to the perceived involvement of the patient in informed medical decision-making (“Did you receive explanations without having to ask for them?” and “Were you involved in medical decision making?”) (ρ = 0.67); and 3) low to moderate correlations between two variables measuring the satisfaction of the information given regarding health status, treatments, investigations and discharge (ρ from 0.41 to 0.64). Four latent variables were created to reflect these correlations in the model (“relationship with nurse”, “relationship with physician”, “perceived involvement” and “satisfaction about information”).

**Fig. 4:**
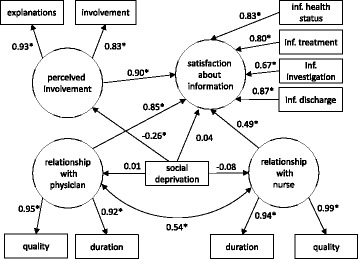
Results of structural equation modelling. Numbers are standardized coefficients, the * indicates coefficients significantly different to zero

Figure 4 presents the final structural equation model and the estimated coefficients. The main determinant of patient satisfaction regarding medical information was the perceived involvement in informed medical decision-making (*p* < 0.001). The qualities of the relationships with the physicians and, to a lesser extent, with the nurses also appeared as significant determinants (respectively *p* = 0.002 and *p* = 0.001). The direct effect of social deprivation on information satisfaction was not significant (*p* = 0.85), but a significant negative relationship between social deprivation and perceived involvement was observed (*p* = 0.001). Overall, this model displayed a very good fit to the data, with a CFI of 0.998 and a RMSEA of 0.022.

## Discussion

Our study shows the existence of a social differentiation in terms of patient satisfaction regarding the medical information delivered by caregivers: socially deprived patients were less satisfied with the information delivered about their health status and treatment, and with the quality and the duration of their interactions with the physician. The patients’ perceived involvement in medical decision-making seemed to play an important role in this social differentiation. Indeed, in our model, this latent factor was strongly and positively linked to the patients’ satisfaction regarding medical information and was negatively linked to social deprivation. The quality and duration of the patients’ interactions with their physicians and nurses also seemed to have a significant impact on their satisfaction.

In previous studies, gender, age, ethnic origin, educational and marital status have all been identified as determinants of patient satisfaction [6, 26, 27], but the analyses of the impact of socioeconomic indicators were frequently inconclusive [6, 28]. A few studies, although, suggested that a lower socioeconomic status could be correlated with a lower satisfaction. Twenty years ago, Davis et al. [29] found that the rates of dissatisfaction with managed care were the highest among lower socioeconomic statuses and minorities. A more recent study in the field of primary care concluded that the most important element of patients’ overall satisfaction was the quality of the communication with their doctor and also observed that patients living in deprived areas were usually less satisfied [30]. Our results also suggest a central role of the physician. Indeed, socially deprived patients were less satisfied with the information exclusively delivered by the physicians, i.e. information regarding their health status and their treatment.

Willems et al. [12] explored the influence of patients’ socioeconomic status and physician-patient communications and showed that patients from lower social classes were given significantly less information and directions. Our study supports these results, showing that patients’ perceived satisfaction about the quality and the duration of physician interactions is significantly lower for deprived patients. Vulnerable patients seem to be doubly penalized: patients from lower social class communicate less actively and express fewer information needs, while concurrently, physicians seem to display more patient-centered communication with patients who are perceived as better communicators, and who expressed positive affect [3]. Moreover, several authors demonstrated that positive physician-patient interaction and communication seem to be facilitated when physicians see themselves as close to their patients in terms of socioeconomic identities and values [3, 7, 14].

Finally, our findings indicate that socially deprived patients feel more frequently that they are not involved in medical decision-making. Krupat et al. [31] showed that patients with a lower educational level were less interested in participating in medical decision-making. It seems therefore that vulnerable patients are caught in a vicious circle affecting their healthcare trajectory: first of all because of their more passive communication style and secondly because of the caregivers’ misperception of their expectations and need for information. This social differentiation in medical information access leads in turn to health inequalities by omission, in the particular context of hospitalizations.

Our study was based on an original design: contrary to most published papers focusing on primary care and on doctor-patient interaction, we explored secondary care and included the perception of nurses. However, this study had a number of limitations. First, we obtained data on 76% of the eligible patients: this raises the possibility of a selection bias. An additional limitation is the potential for measurement error, especially a social desirability bias due to face-to-face interviews with patients and caregivers. Moreover, the EPICES score used for the detection of deprivation has been developed for primary care and not for hospitalized patients. However, it has already been used for inpatients in previous studies [24, 32] and seems to fit in the context of hospitalization, even if some of the items have to be adapted for elderly patients (for example, the item ‘Have you done any sport activities in the last 12 months’). The EPICES score also has the advantage of being adapted to the French context and of taking into account multiple dimensions of the socioeconomic conditions—including psychological, social, and economic aspects. The binary categorization of social deprivation may also be questioned and some refinements on the notion of social deprivation should probably be considered in order to elaborate interventions that are adapted to every scenario. Lastly, our study does not provide any direct evidence about the quality of care and it is unclear whether these differences in perceptions are associated with real differences in care or outcomes. However, several studies illustrated the strong relationship between low socioeconomic status and poorer health outcomes [16, 19].

## Conclusion

Social inequalities in health are a major challenge in modern healthcare systems. To tackle these inequalities, drivers of successful interventions targeted towards vulnerable groups of patients need to be identified.

First, the detection of social vulnerability during care provision could be improved and/or integrated in the hospital’s computerized information systems. Assessing patients’ objective and subjective state of deprivation, as well as their social history and life course perspective should also become common practice, assuming that it would change the way caregivers relate to their patient.

Furthermore, patients’ participation should be promoted in order to facilitate their empowerment. Golin et al. [33] suggested that vulnerable patients whose physicians facilitate participation in clinical decision-making were more satisfied with their care. Greater patient participation has the potential to improve adherence to treatment and health outcomes.

A last approach would be to offer training in communication techniques to physicians and other healthcare professionals. Medical training often ignores cultural, social and psychological aspects of care. Recently, however, several initial or on-the-job courses for practitioners focusing on these relational aspects have been developed, in particular in the English-speaking world. This dimension is indeed central, and several studies have shown the beneficial effect of communication between patients and healthcare staff on the quality of care [34, 35].

In France, the lack of studies exploring patients’ involvement in medical decisions and the role played by social differentiation creates a critical knowledge gap that should be filled. Further research on these subjects is therefore needed in order to improve patients’ satisfaction and reduce health social inequalities.
